# A Conformal Driving Class IV Flextensional Transducer

**DOI:** 10.3390/s18072102

**Published:** 2018-06-30

**Authors:** Tianfang Zhou, Yu Lan, Qicheng Zhang, Jingwen Yuan, Shichang Li, Wei Lu

**Affiliations:** 1 College of Underwater Acoustic Engineering, Harbin Engineering University, Harbin 150001, China; zhoutianfang@hrbeu.edu.cn (T.Z.); Q.Zhang5@salford.ac.uk (Q.Z.); yuanjingwen@hrbeu.edu.cn (J.Y.); lsc1993@hrbeu.edu.cn (S.L.); 2 Acoustic Science and Technology Laboratory, Harbin Engineering University, Harbin 150001, China; 3 Key Laboratory of Marine Information Acquisition and Security, Harbin Engineering University, Ministry of Industry and Information Technology, Harbin 150001, China

**Keywords:** conformal driving class IV flextensional transducer, low frequency, high power, small size

## Abstract

Class IV Flextensional Transducers (FTs) are the most popular among various FTs used as low-frequency and high power underwater acoustic sources. However, an undeniable fact exists in Class IV FTs is that the resonance frequency of breathing mode regulator used is fairly raised by its longitudinal driver stacks. In this research, a conformal driving Class IV FT in which the driver stacks are kept conformal with its oval shell was proposed aiming at the limitations of conventional driving Class IV FTs described above. The device exhibits competitive Transmitting Voltage Responses (TVRs) but much lower operation frequencies with respect to conventional driving Class IV FTs, through the designs of conformal and segmentally controlled driver stacks. Geometric parameters analysis was carried out extensively by Finite Element (FE) simulations for the design optimizations and then a conformal driving Class IV FT resonating at 510 Hz (45% approximately lower than that of conventional driving Class IV FT with the same shell geometry) was finalized. Subsequently the conformal driving Class IV was fabricated and tested in the anechoic tank experimentally. Good agreements of both FE predictions and experimental results demonstrate its low-frequency and small-size acoustic performance.

## 1. Introduction

It is widely known that the low-frequency, small size and high-power underwater transducers exhibit many potential applications in underwater acoustic domain, such as the long-range detection and tracking, marine environmental monitoring and development, as well as the small underwater target platforms (UUV and AUV) [1,2,3,4]. In particular, Flextensional Transducers (FTs) are a generic type of low-frequency, small-size and high-power underwater transducers, which make use of the displacement amplification mechanism by transferring the extensional vibrations of the driver stacks to the flexural vibrations of the shell configuration [5,6,7,8,9].

The first FTs were built and tested in 1929 at the Anacostia Naval Research Laboratory (NRL), Washington DC, by Harvey C. Hayes, the Director of the NRL Sound Division [10]. It was until the middle-to-late 1950s the FTs were rebirthed and investigated further in Toulis’ work [11]. In fact, the Toulis’ design is roughly identical to that of Hayes, with the exception that the piezoelectric ceramic driver stacks were used instead of the magnetostriction-driven rods in Hayes’s transducer. There’re two other patents in parallel with the inventions of Toulis which are the axisymmetric flying-saucer-shaped FTs proposed by Frank R. Abbott [12] and “dogbone” shell FTs designed by Merchant [13], respectively.

Recently most of the efforts about FTs have been concentrated on the broadband operations due to their intrinsic high mechanical quality factors and subsequently narrow bandwidths [14,15,16,17,18]. Porzio [14] invented a slotted cylinder FT which exhibits much broader bandwidth than conventional driving FTs by coupling the low frequency flexural mode and the higher frequency “breathing” mode caused by slotted cylinders. Chen et al. [16] proposed a broadband FT with lengthened major axis by the theory of multiple-mode-coupling. It was shown that the resonance frequencies of the 3 modes of interest (the fundamental mode, the second-order mode and the membrane vibration mode) can be tuned by lengthening the major axis and coupling subsequently to broaden the bandwidth of the transducer. Furthermore, Osborn et al. [17] designed a FT in which the honeycomb structure replaces the solid shells, with the honeycomb providing the same bending stiffness of the solid shells but less weight, therefore an increased operation bandwidth.

In order to achieve the low-frequency operation of FTs, additional radiation structures have been assembled to the oval shell for larger radiation areas and larger effective mass subsequently, which can ultimately lead to lower operation frequencies both in Refs. [19,20].

Among the various FTs, Class IV FT is a particular type which shows superior performance in terms of low-frequency and high-power property [21,22,23,24]. A Class IV FT currently consists of a shell in the form of elliptical cylinder and active diver stacks inside. Longitudinal vibrations of the driver stack, typically in the form of a bar, can be shifted to a combined flexural vibration mode of the shell which exhibits amplified amplitude motions and decreased resonance frequencies. However, compared with the resonance frequency of the shell itself, a drawback brought by this design is that the resonance frequency of the combined flexural mode is fairly raised by the additional effective stiffness caused by longitudinal driver stack.

To solve the above problems, the conformal driving Class IV FT is proposed. This an enhanced type of FT in which the active driver elements are kept conformal and adhered to the oval shell. It is shown by Finite Element (FE) predictions and experimental test that the resonance frequency of the breathing vibration mode of the conformal driving Class IV FT is obviously lower than that of the conventional longitudinal driven FT of the same dimensions. In addition, the methods of segmented driving and phase control are utilized for optimizing the vibrational amplification and subsequently achieving a higher power performance of the transducer. The design of conformal driving Class IV FT is given in Section 2. Parametric analysis by FE simulations and experimental measurements are presented in Section 3 and Section 4 respectively, followed by the conclusions eventually.

## 2. The Design of Conformal Driving Class IV FTs

**Figure 1:**
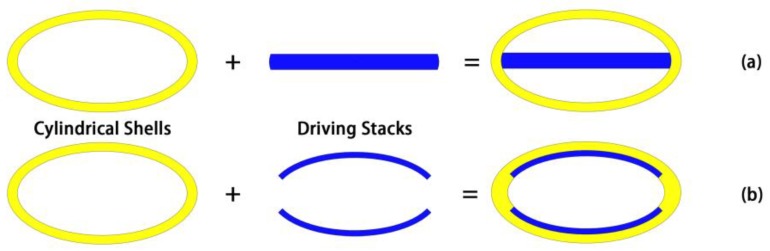
(**a**) The structure of conventional driving Class IV FTs; (**b**) The structure of conformal driving Class IV FTs.

In the conformal driving Class IV FT schematic in Figure 1b, the conformal driver stacks are used instead of the longitudinal driver stacks in conventional driving Class IV FTs plotted by Figure 1a. It is expected that the effective stiffness of the geometry and subsequent vibrational frequency is significantly lowered by the design of conformal driver stacks. Here the conformal driving is defined as a geometric form in which the active driver elements are kept conformal to one or several segments of the shell alternatively. With the conformal driving, more agile and diverse driving algorithms can be adapted to optimize the acoustic performance of FTs. In the following sections, the conformal driving Class IV FT was examined first in regard of its low-frequency performance and then the influences of segmented driving and phase control on its high-power performance. It is critical that the conformal driving in our study is only applicable to the FTs which have cylindrical shells, such as Class IV and VII FTs.

## 3. Finite Element Analysis of Conformal Driving Class IV FT

### 3.1. Modal Analysis

**Figure 2:**
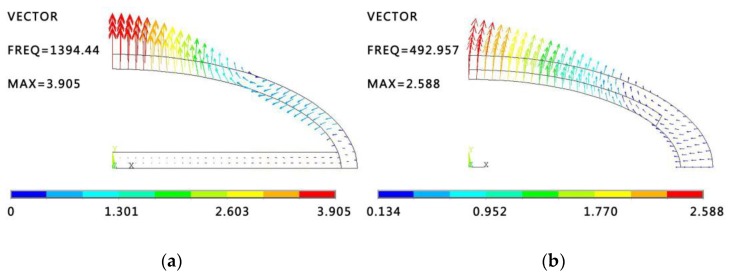
(**a**) The first flexural vibration mode shapes of conventional driving Class IV FT; (**b**) The first flexural vibration mode shapes of conformal driving Class IV FT.

Modal analysis of the conformal driving Class IV FT is carried out with commercial FE software ANSYS. The geometries of conventional driving Class IV FT and conformal driving Class IV FT are shown in Figure 1. Conformal driver stacks (the blue areas) are adhered to the inside of the oval shell to replace the longitudinal driver stacks. Fundamentally, the conformal driver stack is not included in Modal analysis and the shell geometries of these two models are basically the same in the process. The modal shapes of 1/4 model are presented by displacement vector maps in ANSYS Post Processor, as shown in Figure 2.

As can been seen from both figures, there’s a vibration node around the midpoint of the shell at which the displacement equals to zero and the displacements on both sides of the node are anti-phases. This particular mode is defined as a flexural breathing mode (or fundamental flexural mode). The resonance frequency of conventional driving Class IV FT shown in Figure 2a is 1394.4 Hz, while an identical flexural mode resonating at 492.9 Hz is found in conformal driving Class IV FT. An obvious decrease (40% approximatively) of breathing mode frequency of conformal driving Class IV FT is brought out with respect to that of conventional driving Class IV FT, which will witness conformal driving FT benefiting more in the applications of low-frequency and small-size underwater transducers.

### 3.2. Vibrational Displacement Property Analysis

**Figure 3:**
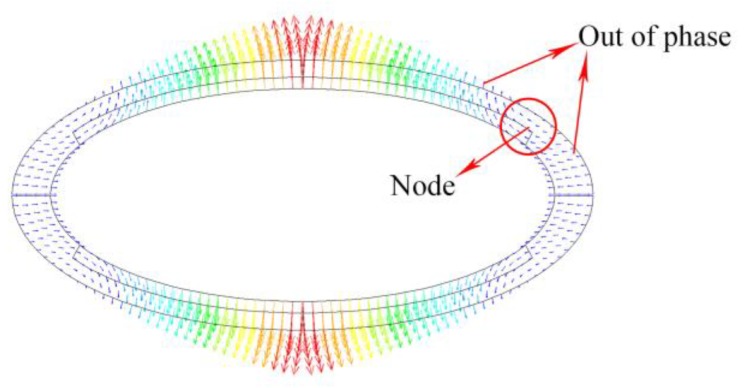
The first flexural vibration mode shapes of conformal driving Class IV FT.

The overall vibrational displacement property of flexural breathing mode of the conformal driving Class IV FT is shown in the Figure 3. Four vibration nodes and reverse displacement distributions are found as described in Section 3.1.

**Figure 4:**
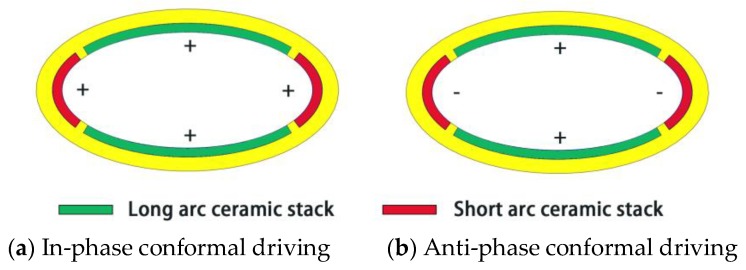
Two types of driving method of conformal driving Class IV FTs.

In order to obtain a superior capability of acoustic radiation power of the transducer, segmented driving and phase control are utilized by taking advantage of the vibrational shape of the breathing mode. To make a comparison, two types of conformal driving method are investigated. Four conformal stacks are assembled in the segments according to the locations of the nodes. The in-phase vibrations and anti-phase vibrations between the adjacent stacks can be excited by controlling the driving voltages. For convenience, we name them as in-phase conformal driving transducer and anti-phase conformal driving transducer respectively, as shown in Figure 4a,b. Thus, the marker “+” represents an expanding vibration and marker “-” represents a contracting vibration. 

**Figure 5:**
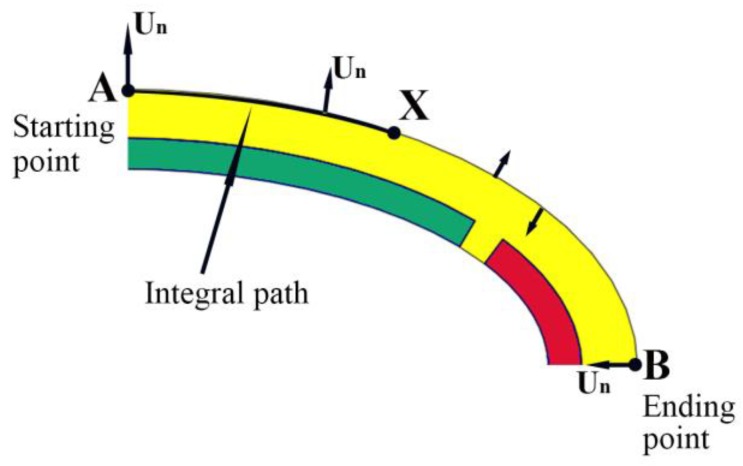
Schematic of volume displacement calculations.

Similar to the acoustic radiation model of pulsating sphere [25], the volume displacement can be used here to quantitatively evaluate the sound radiation capacity of conformal driving Class IV FTs, due to its dimension is much smaller than the wavelength of the sound produced over the frequency range of interest. The volume displacement can be defined as an integral of the normal displacement vector over its radiation surface:

(1) $$X=\underset{s_{0}}{\int }U_{n}\times h\;ds$$

where *S_0_* is the integral distance from minor axis vertex ***A*** to major axis vertex ***B***, *U_n_* is the displacement vector normal to the surface and *h* is the height of shell, as illustrated in Figure 5.

**Figure 6:**
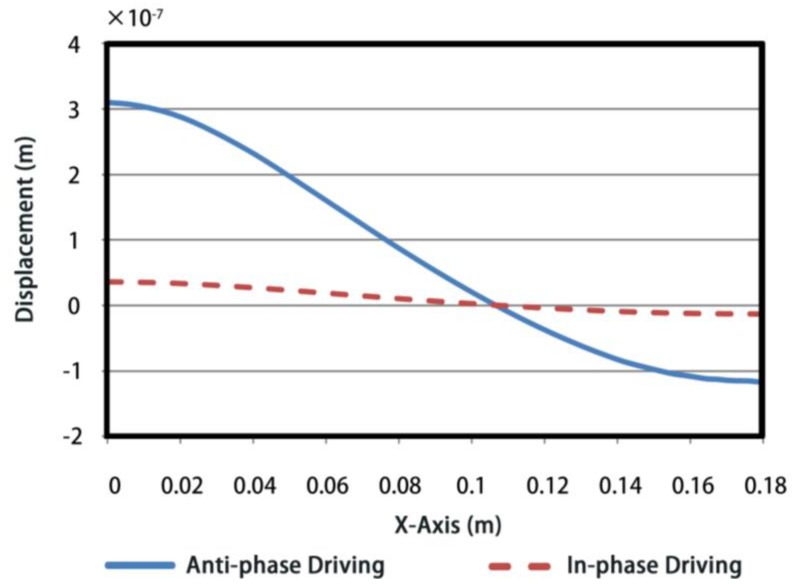
The distributions of the normal displacements along semi-shell of transducer.

**Figure 7:**
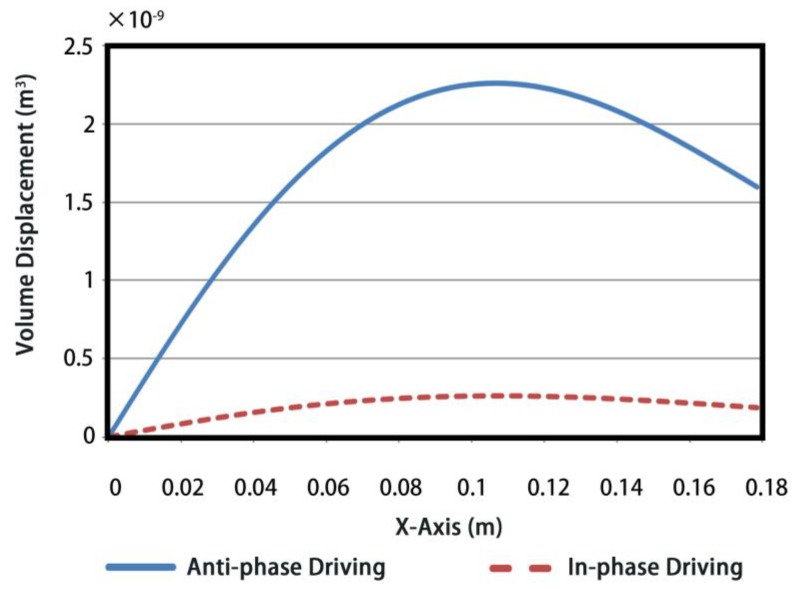
The volume displacements along semi-shell of transducer.

Consequently, the distributions of the normal displacements along semi-shell are obtained by FE simulations, as shown in Figure 6. The values of volume displacements are presented in Figure 7, under 1 Volt excitation voltage. To be specific, x-axis presents the arc distance from minor axis vertex A to the current point X and y-axis is the corresponding accumulation of volume displacement. Note that following analysis of volume displacements is under the same resonance frequency for two driving methods.

As can be seen from Figure 6, the vibration nodes occur at the same points for both types of conformal driving FTs, 0.108 m away from the minor axis vertex. However, the peak displacement of anti-phase conformal driving FT is an order of magnitude larger than that of the in-phase one, even though they have identical vibrational modes.

Similarly, in Figure 7, a volume displacement of 1.59 × 10^−9^ m^3^ is obtained for the anti-phase FT, which is also approximately an order of magnitude larger with respect to the 1.87 × 10^−10^ m^3^ volume displacement of the in-phase one. Both of the results of Figure 6 and Figure 7 illustrate a larger acoustic power. Therefore, a superior performance of acoustic radiation can be realized by segmented driving and anti-phase control.

**Figure 8:**
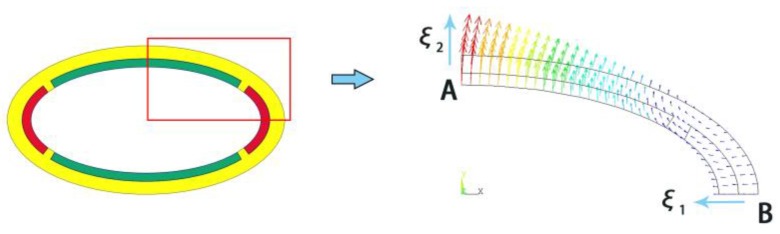
Displacement amplification motion of Conformal Driving Class IV FT.

As is known the conventional driving Class IV FT is most applicable due to its effect of displacement amplification described above. Displacement amplification ratio is used to indicate the vibrational amplification property of Class IV FTs frequently [3,26], which is defined as the ratio of the normal displacement ξ_2_ at the end of minor-axis (point A) to the normal displacement ξ_1_ at the end of major-axis (point B), as illustrated in Figure 8. Here a FE model is performed to examine the effect of the displacement amplification of conformal driving FT. The results show that the displacement amplification ratio can reach 2.7 for the conformal driving FT under the particular size considered above, which claims that the conformal driving FT works equally well in terms of the effect of displacement amplification.

### 3.3. Acoustic Radiation Performance Analysis

**Figure 9:**
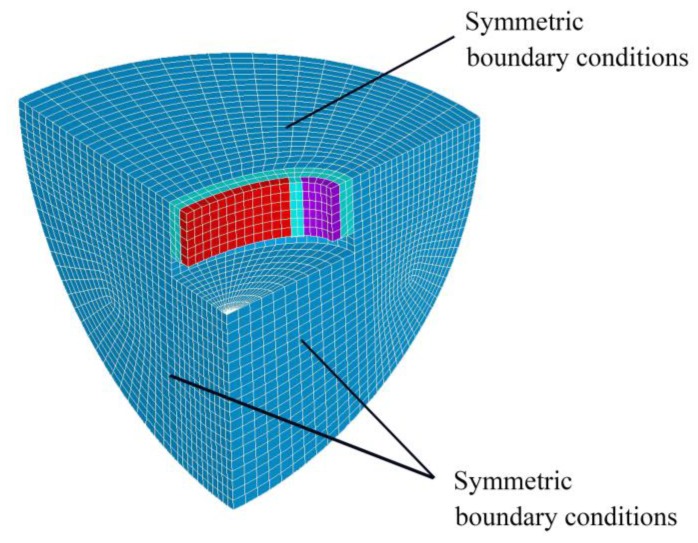
The finite element model of the transducer in water.

The FE model in Figure 9 is constructed in ANSYS to describe the underwater acoustic radiation of the conformal driving Class IV FT. 1/8 of the geometry is modeled and 3 symmetric conditions are applied to improve the simulation efficiency. No loading is applied on the top surface and bottom surface to simulate free boundary conditions caused by soft silicon layer. We assign the piezoelectric ceramics by Solid5 element, aluminum shell by Silod45, water area by Fluid30 and outermost water layer by Fluid130 to simulate the nonreflecting boundary. A water area of 0.8 m radius is constructed for far-field conditions. Hexahedral mesh grids are structured with 0.01 m mesh size. The meshing cells add up to 57483. The epoxy adhesive layer, silicon rubber layer, top cover plate and bottom cover plate used in the fabrication are neglected in FE model.

The transmitting Voltage Response (TVR) of transducer is calculated by Harmonic Analysis in ANSYS:

TVR = 20 × log(*p(r)* × r / *p_ref_*) (2)

where *p*(*r*) is the far-field pressure generated at r meter range by the transducer in water, which can be collected in FE model directly. *P_ref_* = 1 μPa/V is reference pressure. Parametric analysis is carried out to investigate the sole effect of each structural parameter through single-variable method. The structural parameters we considered including the length of major axis, the ratio of major-minor axis, the thickness and height for the oval shell, as well as the number and the height of the conformal driver stacks.

**Figure 10:**
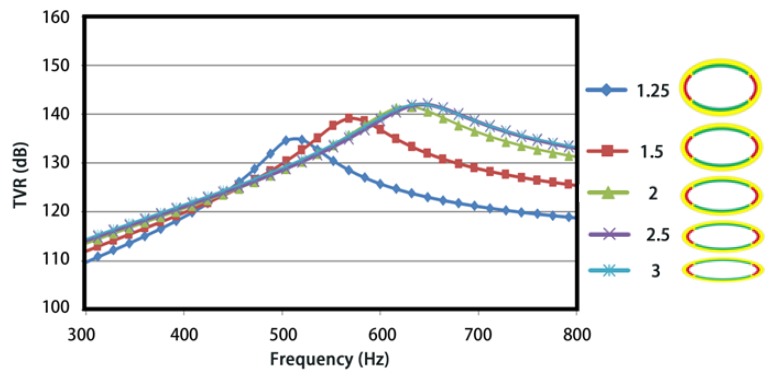
Influence of the ratio of major-minor axis on the TVR.

The influence of the ratio of major-minor axis under a constant length of major axis was investigated first. TVRs are plotted in Figure 10, from 300 Hz to 800 Hz. 5 curves with different markers represent the TVRs under various ratios of major-minor axis. On can note that the first-order resonance frequency (the peak in curves) rises with the increasing ratio from 1.25 to 2.5 and approaches to a constant when the ratio reaches 2.5. Similar variation patterns are found for TVR values at the resonance frequencies. The variations of resonance frequency are attributed to the increase of effective stiffness and the decrease of effective mass of the FT caused by a shorter minor axis. As for the variations of TVRs, a reduced region of anti-phase and subsequent greater acoustic radiations (or TVR) are obtained with the node’s movements toward major axis for a shorter minor axis. From another point of view, a higher resonance frequency represents a larger radiation resistance on the same radiation area and therefore a greater acoustic radiation performance.

**Figure 11:**
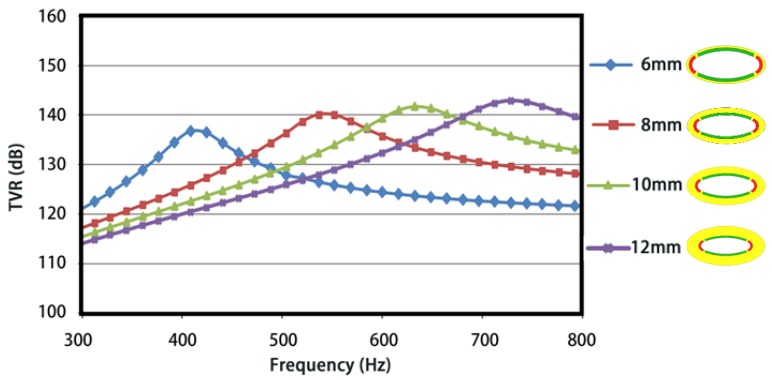
Influence of the shell thickness on the TVR.

Figure 11 presents the numerically simulated TVRs for the conformal driving FTs with various shell thicknesses. An obvious variation pattern was seen that the first-order resonance frequency and the corresponding TVR go up along with the increase of shell thickness. It can be explained simply that a thicker shell has a larger effective stiffness and therefore a higher resonance frequency, subsequently a larger radiation resistance and higher TVR under unchanged radiation areas.

**Figure 12:**
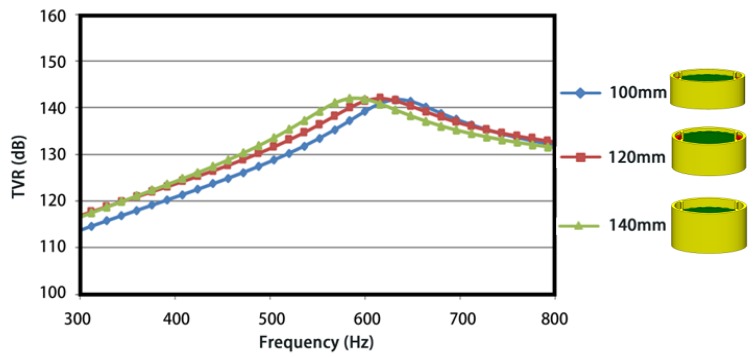
Influence of the Shell height on the TVR.

**Figure 13:**
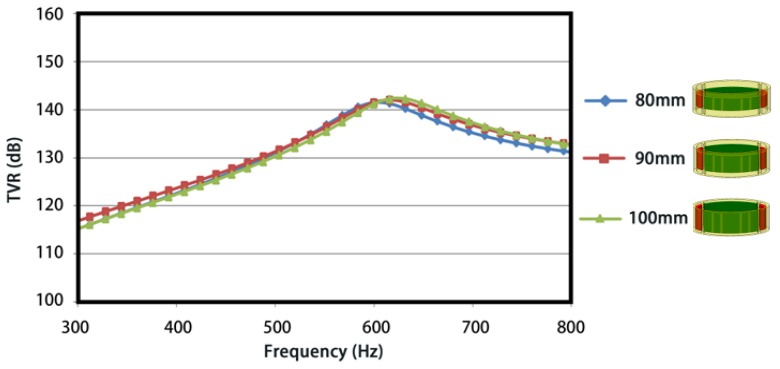
Influence of the driver stacks height on the TVR.

In Figure 12 and Figure 13, the simulation shows that both the heights of oval shell and driver stacks of conformal driving FT make little influence on its acoustic radiation performance. Without loss of generality, the resonance frequency goes up with the height of shell and goes down with the height of driver stacks. While the TVRs hold steady with the height of shell and increase slightly with the height of driver stacks.

**Figure 14:**
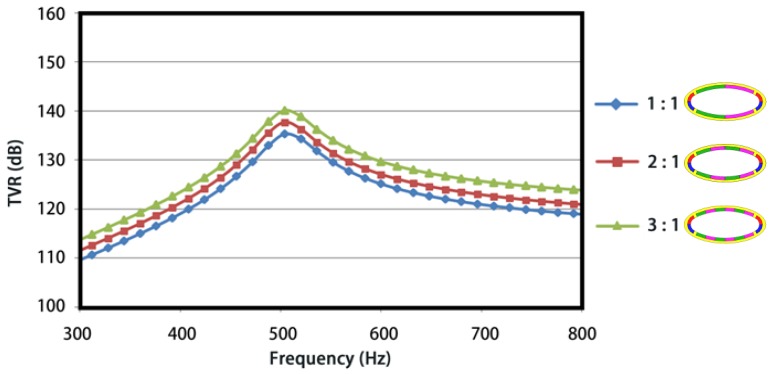
Influence of the ratios of ceramics’ number in long and short arc stacks on the TVR.

At last, the ratios of the number of ceramics in long and short arc piezoelectric ceramic stack are considered, while the number of ceramic pieces in short arc stack is fixed. According to the results plotted in Figure 14, no effect has been found from the ratios on the resonance frequency, while an increase of TVRs can be seen in the case of more piezoelectric ceramics in long arc stacks. Therefore, for a greater acoustic radiation performance, more ceramics are assembled as possible in long arc stacks in the fabrication process. 

The analysis of structural parameters above gives a way to determine the accurate geometry of the conformal driving Class IV FT with a view to obtain an optimal performance. Generally, the major-minor axis ratio and shell thickness have greater impact on the in-water resonance frequency of the transducer and therefore are treated as major adjustable factors. While the heights of oval shell and driver stacks make a lesser influence on resonance frequency and are used for fine tuning. The in-water TVR is expected to be modified by the shell height and the numbers of piezoelectric ceramics in long and short arc stacks. Attention should be paid to the fact that a larger major-minor axis ratio causes a higher in-water resonance frequency for conformal driving FT, while conventional driving FTs take the opposite tack. It is an outstanding point that makes the conformal driving Class IV FT preferable candidates for regular-geometry, small-size and high-power acoustic sources.

**Figure 15:**
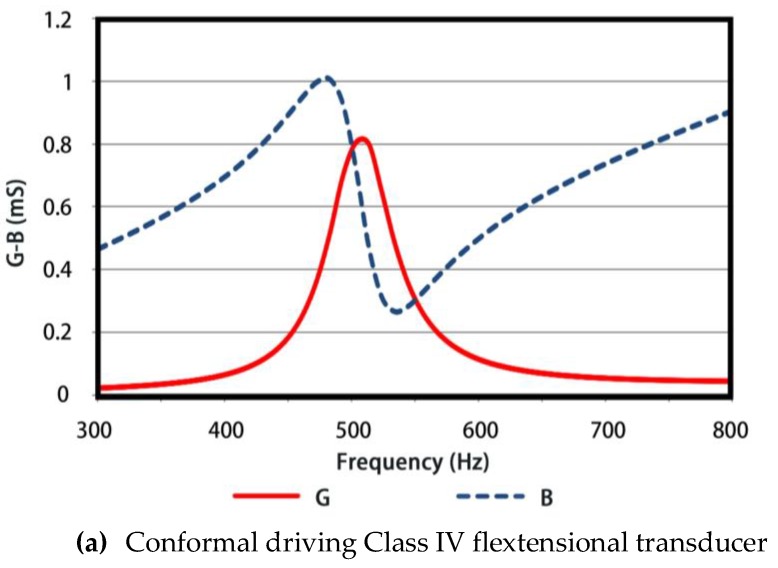

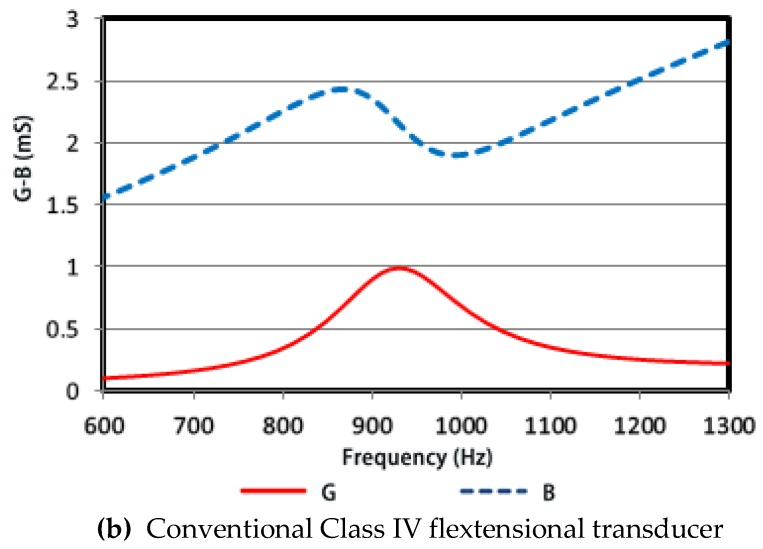
The admittance curves of two types of Class IV FT in water.

A particular conformal driving Class IV FT was investigated based on the analysis above. The structural parameters of the transducer are listed in Table 1. The in-water Admittance vs Frequency plot of FT obtained by FE simulations is shown in Figure 15a. It can be seen that the breathing resonance reaches 510 Hz and the conductance value wherein is 0.8 mS. As comparisons, a conventional Class IV FT (10.4 kg in weight in simulations) of same dimensions is examined, whose structural parameters are list in Table 2. The resonance frequency is 940 Hz and conductance in-water is 1 mS, as illustrated in Figure 15b.

**Figure 16:**
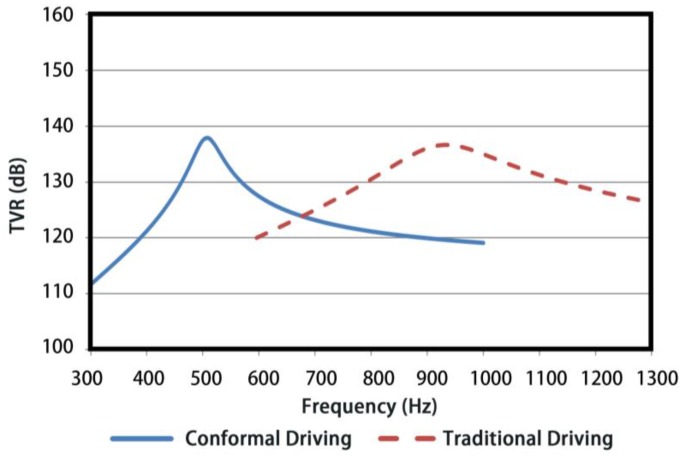
TVRs of the conformal and conventional driving Class IV FT.

The in-water TVRs of two types of Class IV FTs are plotted in Figure 16, wherein a maximum value of 137.5 dB at resonance frequency 510 Hz for conformal driving Class IV FT is presented by blue solid line, in comparison with the conventional one of same shell geometry (red dotted line) whose peak TVR is 136 dB locating at 940 Hz. The significant fall of resonant frequency (45% approximately) and slight change of TVR for conformal driving Class IV FT demonstrate its advantages in low-frequency underwater applications.

## 4. Fabrication and In-Water Testing of Conformal Class IV FT

**Figure 17:**
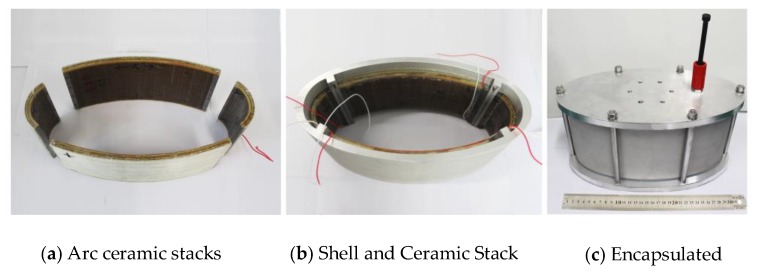
Photographs of the Conformal Driving Class IV FT at different stages of assembly.

A conformal driving Class IV FT schematic in Figure 17 was fabricated based on the FE optimization above. The conformal driver element is composed of 4 sets of spliced arc-shaped piezoelectric stacks, adhering to the inside of the shell. The arc-shaped piezoelectric ceramics are PZT4 customized by Haisheng Tech. Company (Yichang, China) [27] and the oval shell is aluminum. The oval shell is clamped by the top and bottom cover plate and jointed by silicon rubber gaskets forming an air backing geometry. The driver stacks are pre-stressed by the oval shell and the epoxy glass layers attached to the stacks are used for higher mechanical strength and electric insulation. Materials properties of all components are listed in Appendix A. The dimensions of conformal driving Class IV FT finalized are 0.33 m long, 0.17 m wide and 0.15 m high and 9.2 kg in weight.

**Figure 18:**
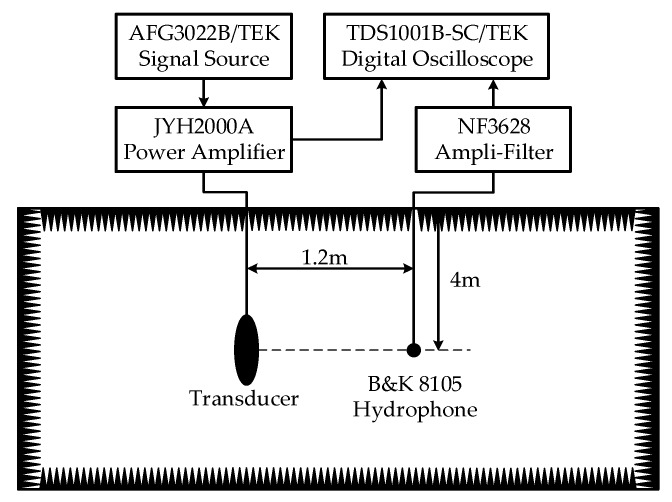
Diagram of testing system.

**Figure 19:**
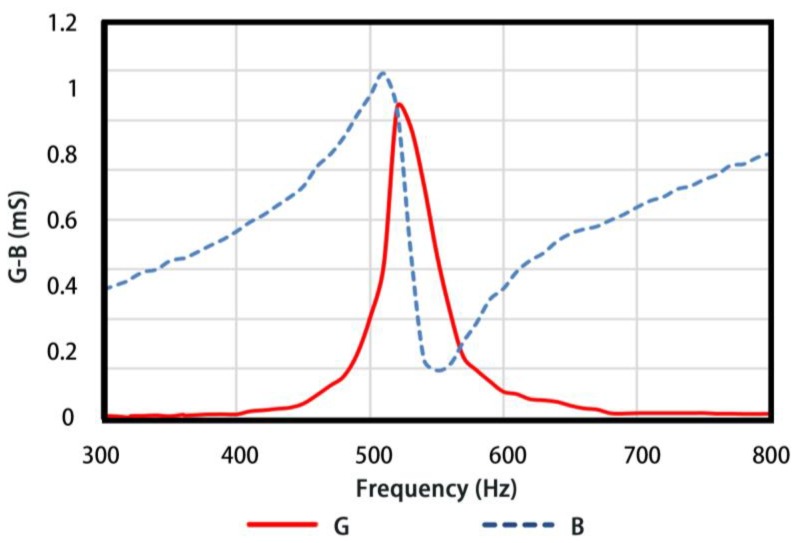
Admittance curves of conformal driving transducers in water.

The transducer is tested in the anechoic tank (25 m × 15 m × 10 m) in Key Laboratory of underwater acoustic science and technology, Harbin Engineering University, Harbin, China. The diagram of the testing system is presented in Figure 18. The transducer is tested at a depth of 4 m underwater. A distance of 1.2 m is set between the transducer and the hydrophone to ensure far-field conditions. CW pulse signal is chosen as excitation to reduce sound reflections from the sidewalls of tank. In-water admittance curves are presented in Figure 19. The resonance frequency is 520 Hz and the corresponding conductance is 0.62 mS.

**Figure 20:**
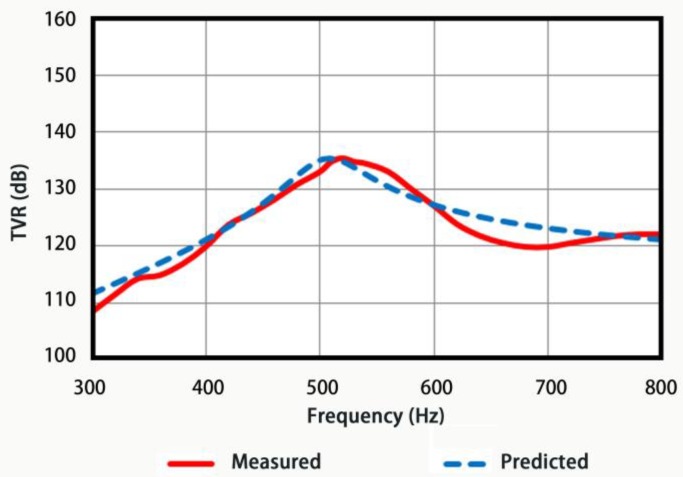
The transmitting voltage response curves of the transducer in water.

Figure 20 shows both the TVR measured experimentally (red solid line) and TVR predicted by FE simulations (blue dotted line) of the transducer. The comparisons between them are listed in Table 3. Both of them are in good agreement in terms of resonance frequencies, while the little discrepancies of TVRs and conductance can be explained by the insufficient bonding strength, which is caused by unmatched curvature radius between the driver stacks and oval shell in the fabrication process.

## 5. Conclusions

A new type of Class IV FT, conformal driving FT has been proposed as a means to improve the low-frequency performance of conventional FTs. In particular, a transducer resonating at 520 Hz and owning 135.4 dB TVR was fabricated and experimentally characterized based on FE simulations. Both FE predictions and experimental testing prove that a significant decrease of resonance frequency and almost unaffected TVRs can be obtained by the design of conformal driving. It provides the conformal driving Class IV FTs with many potential applications as low-frequency, small-size and high-power underwater acoustic sources. Several specific conclusions are summarized as the follows:

1. The conformal driving is proposed as a new type of driving geometry, which can significantly lower the resonance frequency of Class IV FT and enrich its structural design diversity.
2. Segmented driving and anti-phase control are utilized to the conformal driving Class IV FT by matching the vibrational shape and displacement nodes of the oval shell, which can maintain the high-power operation of the transducer in parallel to the displacement amplification of conventional FT.
3. 45% decrease of resonance frequency of conformal driving Class IV FT is presented with the shell geometry unchanged both by FE predictions and experimental measurements.

Last but not least, deeply analyzing and mining the superiority of conformal driving Class IV FT are supposed to be done in further work. There are some fabrication technologies to be improved, such as the precision controls of the conformal driver stacks, the pre-stressing process and so forth.

## Figures and Tables

**Table 1:** Structural sizes of Conformal Driving Class IV FT.

Parameter	Value	Parameter	Value
Length of major axis of the shell	300 mm	Height of the piezoelectric ceramic stack	80 mm
Length of minor axis of the shell	140 mm	Thickness of the piezoelectric ceramic stack	7 mm
The average thickness of the shell	10 mm	Number of Long arc piezoelectric ceramic stack	14 pieces
Shell height	120 mm	Number of Short arc piezoelectric ceramic stack	42 pieces

**Table 2:** Structural sizes of conventional Class IV FT.

Parameter	Value	Parameter	Value
Length of major axis of the shell	300 mm	Shell height	120 mm
Length of minor axis of the shell	140 mm	piezoelectric ceramic stack	182 mm (L) × 30 mm (W) × 80 mm (H)
The average thickness of the shell	10 mm	Number of piezoelectric ceramic stack	52 pieces

**Table 3:** Comparisons of predicted values to those measured on the prototype.

Item	Resonance Frequency in Water/Hz	Conductance of the Resonance /mS	Maximum Value of TVR/dB
Ansys model	510	0.80	137.5@510 Hz
Experimental	520	0.62	135.4@520 Hz
Difference: model vs. experimental	10	−0.18	−2.1

## Appendix A

Material properties

1. Aluminum:

Density: 2790 kg/m**^3^**, Young’s modulus: 71.5 GPa, Poisson’s ratio: 0.34.

2. Silicon rubber:

Density: 1094 kg/m**^3^**, Young’s modulus: 311 MPa, Poisson’s ratio: 0.48.

3. Epoxy resin:

Density: 1250 kg/m**^3^**, Young’s modulus: 3.6 GPa, Poisson’s ratio: 0.35.

4. Piezoelectric ceramic (PZT-4):

**Table A1:** Material properties of piezoelectric ceramic (PZT-4).

**C_11_^E^** **(10^10^ N/m^2^)**	**C_12_^E^** **(10^10^ N/m^2^)**	**C_13_^E^** **(10^10^ N/m^2^)**	**C_33_^E^** **(10^10^ N/m^2^)**	**C_44_^E^** **(10^10^ N/m^2^)**	**C_66_^E^** **(10^10^ N/m^2^)**
13.9	7.78	7.43	11.5	2.56	3.06
**Density (kg/m^3^)**	$\varepsilon_{11}^{s}/\varepsilon_{0}$	$\varepsilon_{33}^{s}/\varepsilon_{0}$	$e_{31}$ **(C/m^2^)**	$e_{33}$ **(C/m^2^)**	$e_{15}$ **(C/m^2^)**
7500	730	635	−5.2	15.1	12.7
